# Neonatal Outcome following External Cephalic Version (ECV)—Comparison between Vaginal Birth after Successful ECV and Elective Caesarean Section after Unsuccessful ECV

**DOI:** 10.3390/jcm13133837

**Published:** 2024-06-29

**Authors:** Felix Borgmeier, Sophia Horst de Cuestas, Maximilian Pruss, Noa Fath, Carsten Hagenbeck

**Affiliations:** 1 MVZ Amedes for Prenatal-Medicine und Genetic GmbH, 40210 Düsseldorf, Germany; f.borgmeier@praenatal.de; 2 Department of Psychiatry, Psychotherapy and Psychosomatics of Children and Adolescents, University Hospital Rheinisch-Westfälische Technische Hochschule Aachen, 52074 Aachen, Germany; shorstdecues@ukaachen.de; 3 Department of Obstetrics and Gynecology, University Hospital Düsseldorf, 40225 Düsseldorf, Germany; maximilian.pruss@med.uni-duesseldorf.de (M.P.); noa.fath@med.uni-duesseldorf.de (N.F.)

**Keywords:** breech presentation, external cephalic version, caesarean section

## Abstract

**Introduction**: In 3–6% of pregnancies, foetuses can be expected to be in a breech presentation near term. Consultation concerning further management of the pregnancy, including the option of an external cephalic version (ECV), is recommended by international guidelines (RCOG, ACOG, and DGGG). With regards to an ECV, there need to be two assumptions. Firstly, the procedure is safe, which has been shown adequately. Secondly, a vaginal birth after a successful ECV needs to prove to be non-inferior to the alternative of an elective caesarean section. The aim of this study is to assess the non-inferiority assumption. **Methods**: Overall, 142 singleton pregnancies were analysed that presented a foetus in a non-cephalic presentation and underwent an ECV near term between 2011 and 2020. The ECV was performed at 36 weeks of gestation for primiparous women and at 37/38 weeks of gestation for multiparous women. To assess neonatal outcome, the following parameters were recorded: arterial and venous umbilical cord blood pH, APGAR scores and admission to the neonatal intensive care unit (NICU). Data were analysed under the assumption that neonatal outcome does not differ between elective caesarean sections with or without an ECV in advance. **Results**: The success rate of an ECV was 56.3% (80/142). In the case of a successful ECV, there was a 77.5% (62/80) chance for a vaginal delivery. The mean arterial pH for neonates born vaginally after successful ECV was 7.262 (SD 0.089), compared to 7.316 (SD 0.051) for those born via elective caesarean section (*p* < 0.001). APGAR scores at 1, 5, and 10 min were similar between the groups, with a slightly higher proportion of neonates scoring below the median in the caesarean section group. Specifically, 13.7% (7/51) at 1 min, 15.7% (8/51) at 5 min, and 9.8% (5/51) at 10 min in the caesarean section group were below the median, compared to 4.92% (3/61), 4.92% (3/61), and 3.28% (2/61) in the vaginal birth group. NICU admission rates were 3.28% for vaginal births and 3.92% for elective caesarean sections (*p* > 0.05). **Conclusions:** Women with a successful ECV can expect a neonatal birth outcome after a vaginal birth that is non-inferior to an alternative elective caesarean section.

## 1. Introduction

The incidence of a breech presentation at term is approximately 3–6% [1,2,3]. The optimal approach for managing breech presentations is often associated with uncertainties in daily hospital practice. One of the most influential publications is the Term Breech Trial [3]. Despite several follow-up studies, a vaginal breech birth remains relatively risky when compared to a planned caesarean section from a breech presentation. The subsequent drop in vaginal breech births is substantial [4]. An external cephalic version (ECV) poses an easy and non-invasive opportunity that can enhance the chance of a vaginal birth from a cephalic presentation [1]. Despite official recommendations from international guidelines to perform an ECV in the absence of absolute contraindications, many expectant parents and obstetricians express doubts about the benefits of attempting an ECV [5]. Even though most scientific data indicate the ECV procedure to be safe, questions remain concerning the risks of labour itself and early neonatal period [6].

First and foremost, we posit that giving birth from a cephalic presentation following a successful ECV is non-inferior to the alternative of an elective caesarean section (ES) from a breech presentation. To substantiate this hypothesis, we compared a sample of singleton term pregnancies with foetuses in a breech presentation delivered by ES with a sample that achieved vaginal birth following a successful ECV.

## 2. Methods

### 2.1. Study Set-Up

**Figure 1:**
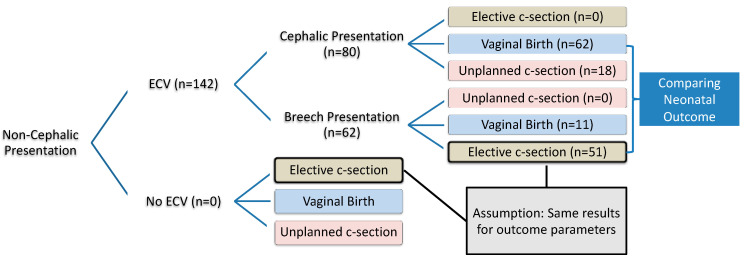
Study set-up and possible paths of clinical course of action.

Figure 1 shows the study set-up. This retrospective cohort study aimed to assess the non-inferiority of vaginal birth after successful ECV compared to elective caesarean section. All singleton pregnancies that underwent an ECV attempt and subsequent delivery at the University Hospital for Women in Duesseldorf, Germany, between 2011 and 2020 were included. The ECV was performed at 36 weeks of gestation for primiparous women and at 37/38 weeks of gestation for multiparous women.

### 2.2. ECV Procedure

The entire ECV was undertaken as a day-case procedure. Upon arrival, the expectant mother was given an intravenous cannula and underwent standard foetal and maternal monitoring, including vital parameters, CTG, and foetal sonography, which included foetal Doppler ultrasound of the umbilical artery (A. umbilicalis) and middle cerebral artery (A. cerebri media: MCA). An obstetric surgical team was prepared to intervene if necessary. With the patient in a supine position, a lubricant was applied liberally on the abdomen to reduce friction during the manoeuvre. To improve disengagement, the woman was asked to take up a slight Trendelenburg position. Initially, the foetal breech was carefully mobilized to allow the foetus to disengage from the maternal pelvis. The physician then encouraged the foetus to perform a forward or backward roll by gentle guidance of the foetal breech and head. The foetal heart rate was intermittently monitored using ultrasound during the ECV manoeuvres. Each ECV procedure involved up to three attempts. If unsuccessful, a second attempt was considered. Tocolytic agents were optional and could be used if ECV appeared challenging [7,8]. No anaesthesia was used during the ECV procedures. Conducting a one-hour CTG after the procedure and 4 hours later assessed foetal wellbeing. Additionally, an experienced sonographer assessed the placenta using ultrasound to exclude any hematoma and monitored foetal well-being with foetal Doppler ultrasound of the umbilical artery and MCA. In the absence of abnormalities, the expectant mother was discharged. Routine obstetric checks were conducted in cases of a successful version. Subsequent obstetric interventions were undertaken according to German guidelines [9,10].

In cases of an unsuccessful ECV trial, an elective caesarean section (ES) was scheduled 7 to 10 days before the due date. For caesarean sections, we routinely use a gentle, modern modified Misgav Ladach technique in accordance with German guideline [11]. The procedure begins with patient preparation and spinal anaesthesia. A transverse skin incision (Pfannenstiel incision) is made 2–3 cm above the symphysis pubis, followed by a transverse fascial incision and blunt dissection of the rectus muscles. The abdominal cavity is accessed by separating the layers without cutting. A low transverse uterine incision is made to deliver the baby. The umbilical cord is allowed to pulsate for at least 60 s before clamping. The uterine incision is closed in a single layer and the fascia and skin with a continuous suture. If the baby is in good condition, it is placed on the mother’s chest immediately after delivery, even while the caesarean section is still ongoing. Postoperative care includes monitoring for complications, pain management, and breastfeeding support.

### 2.3. Data Collection

Collected data were entered into an Excel spreadsheet for statistical analysis via Excel and SPSS. The following information was recorded to allow data analysis pseudo-anonymously:

- Maternal Factors: Pseudonym, age, weight, size, BMI, gravity, parity, placental location, ECV outcome, use of fenoterol intravenously, gestational age at ECV attempt and delivery, mode of delivery.
- Foetal Factors: Presentation at ECV attempt, sex, arterial and venous pH, base excess, admission to neonatal intensive care, 1-, 5-, and 10-min APGAR scores, weight at birth, length at birth, head circumference at birth.

### 2.4. Objectives

Our primary objective was to assess early postnatal outcome in terms of mortality and morbidity rates. Morbidity was assessed using neonatal hospitalization and arterial and venous pH, as well as 1-, 5-, and 10-min APGAR scores. Secondary outcome parameters included success rates of ECV, rates of unplanned caesarean sections, and rates of assisted deliveries. Statistical significance was assumed at an alpha-value below 0.05 and *p*-values were included whenever meaningful.

Due to imprecise coding of procedures, it was impossible to obtain a reliable sample of pregnancies in a breech presentation that had an ES without an ECV trial in advance (Figure 1 “No ECV” (*n* = 0)). Hence, to answer the study question, the assumption needed to be made that an unsuccessful ECV would have no adverse effect on the neonatal outcome after an ES. Therefore, the assumption was that the neonatal outcome is the same for foetuses delivered by caesarean section from a breech presentation with prior exposure to an external version trial compared to foetuses being delivered by caesarean section from a breech presentation without a previous ECV trial (see Assumption in Figure 1).

## 3. Results

### 3.1. Structuring Study Sample

The observational retrospective single-centre study conducted at the Department of Obstetrics of the University Hospital for Women included 152 singleton pregnancies with foetuses in a non-cephalic presentation between the 36th and 37th week of gestation. Data collection commenced in 2011 and concluded in August 2020. All participants met the current national medical guidelines for eligibility to undergo an ECV to facilitate an attempt at spontaneous vaginal delivery. Ten cases were excluded due to significant gaps in the collected data, resulting in 142 cases that were subjected to detailed data analysis. Overall, the success rate for attempted ECV was 56.3% (80/142). Following a successful ECV, the likelihood of achieving a vaginal birth was 77.5% (62/80). In the vaginal birth group, 91.9% (57/62) of deliveries were spontaneous, while 8.1% (5/62) required vacuum extraction. A trial for labour following a successful ECV resulted in an unplanned caesarean section in 22% (18/80).

Conversely, 43.7% (62/142) of the cases had an unsuccessful ECV, with 82.2% (51/62) subsequently undergoing a planned caesarean section. The remaining 17.8% (11/62) delivered spontaneously, although the foetal presentation was unknown; therefore, these cases were excluded from the analysis.

A trial of ECV resulted in a vaginal birth in more than 50% (73/142) of cases, with 85% (62/73) of these from a known cephalic presentation. Additionally, 35.9% (51/142) underwent a planned caesarean section due to an unsuccessful version. Conversely, in the absence of an ECV trial, 17.3% can expect a vaginal birth (82.3% (51/62) ES rate), assuming the proportions of vaginal births are consistent with those observed in this study.

The absolute and relative proportions of the delivery modes are presented in Table 1. There were no ESs after a successful version, as the expectant mother intended to attempt a vaginal birth. The absence of unplanned caesarean sections following an unsuccessful version is likely due to inconsistencies in data collection. In some cases, labour may have commenced prior to the planned caesarean section. However, the birth outcomes can be expected to be similar, as labour would be interrupted immediately upon admission.

### 3.2. Describing Study Sample: Basic Sample Characteristics

Table 2 outlines the basic maternal characteristics of both study groups. With respect to maternal height, weight, and BMI, both groups appear to be homogenous. However, there is a statistically significant difference in maternal age. Parity also has a significant impact on the mode of delivery, as illustrated in Table 3. The odds ratio for achieving a vaginal birth after a successful ECV is 5.73 [95% CI 2.73–12.04] times higher in multiparous women compared to nulliparous women, indicating a significantly greater likelihood of vaginal delivery in multiparous women. 

**Figure 2:**
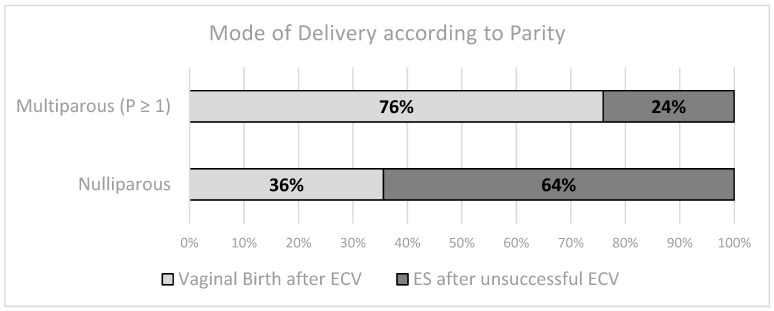
Depicting proportions of the mode of delivery according to parity. In the case of nulliparous women, the mode of delivery seems to be predominantly ES. The opposite is the case for multiparous women, with them being more likely to succeed in a vaginal birth.

For nulliparous women, the likelihood of receiving an ES is nearly twice as high (38/59, 64%) compared to undergoing a vaginal birth (21/59, 36%), excluding the risk of an unplanned caesarean section. Conversely, for multiparous women, achieving a cephalic vaginal birth, given a breech presentation near term, is more feasible (see Figure 1 and Figure 2).

Table 4 summarises basic neonatal parameters, showing very similar values for neonatal length and neonatal head circumference, approximately 50 and 35 cm, respectively. Neonates born vaginally tend to be older by roughly a week and heavier by just under 200 g, on average. 

The sample variance for gestational age and birth weight is also greater in the vaginal birth group. These observations are confirmed by statistical significance, as shown in Table 5.

### 3.3. Comparing Neonatal Outcome Parameters (pH und APGAR)

In both samples, two neonates (3.28% after vaginal birth vs. 3.92% after an ES) required admission to the neonatal ICU (*p* > 0.05).

**Figure 3:**
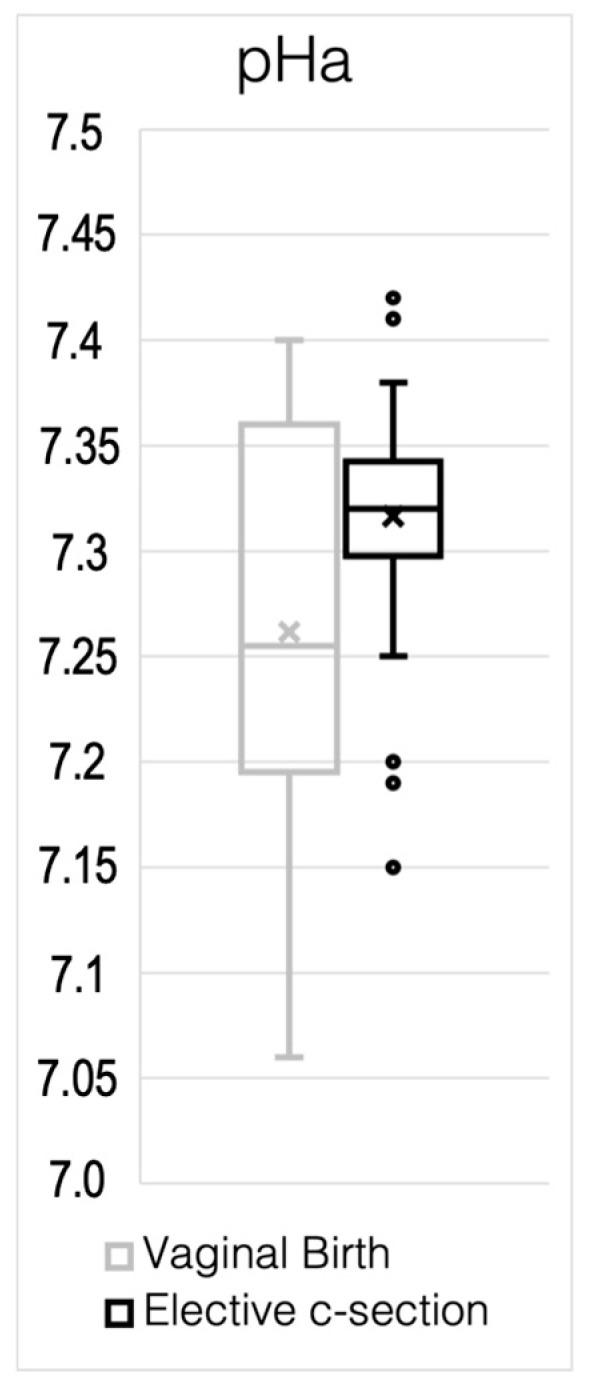
Both sample distributions for postnatal arterial pH, depicting the description from Table 6.

Comparing arterial pH between both groups shows a clear difference in mean and standard deviation, as demonstrated in Figure 3. Table 6 illustrates major differences in the basic statistical parameters describing both samples. On average, neonates born vaginally have a much lower arterial pH of 7.26 compared to 7.31. Additionally, there appears to be a much wider spread of data in the vaginal birth sample, as seen in Figure 3. Expectedly, the standard deviation in the vaginally born sample is larger than in the ES sample, resulting in a substantial difference in the standard error of the mean, being nearly twice as high in the vaginally born sample. Hence, there is a significant statistical difference between both sample distributions (Table 6), assuming a normal distribution.

**Figure 4:**
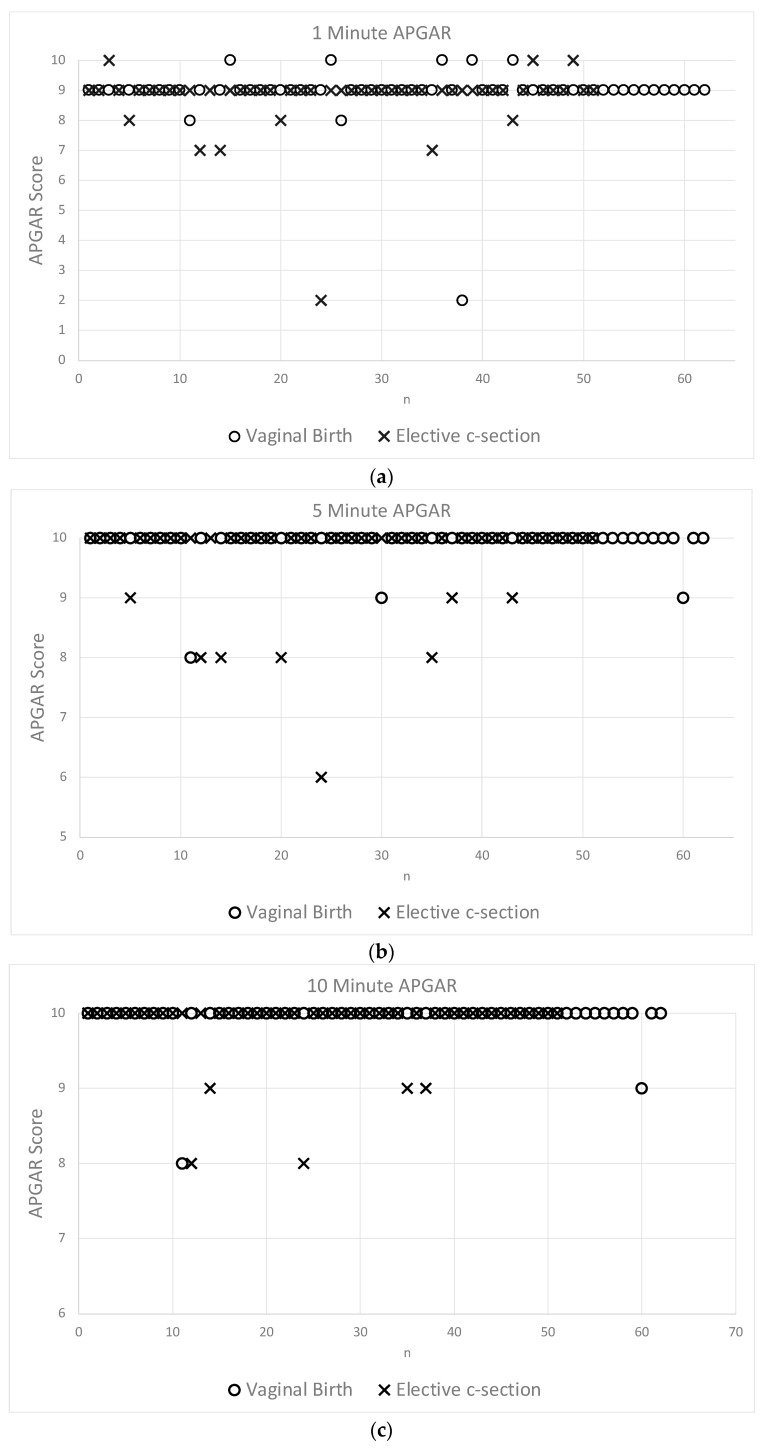
(**a**–**c**) 1-, 5-, and 10-minute APGAR scores for each individual. Neonates after a vaginal birth, marked with an “o”, are less often below the median than neonates after an ES, marked with an “×”.

Figure 4a–c display individual APGAR scores for the 1st, 5th, and 10th postnatal minute. The crosses represent the ES sample and the circles represent the neonates born vaginally. Medians for both samples in the 1st, 5th, and 10th minute are 9, 10, and 10, respectively. It appears that in all of Figure 4a–c, there are more neonates below the median after an ES. Specifically, there are 13.7% (7/51 at 1 min APGAR), 15.7% (8/51 at 5-min APGAR), and 9.8% (5/51 at 10 min APGAR) below the median after an ES compared to 4.92% (3/61), 4.92% (3/61), and 3.28% (2/61) after a vaginal birth, including five vacuum extractions. 

With regards to the prognostically valuable 5-min APGAR score, there are twice as many neonates below the median after an ES than after a vaginal birth. A Chi-squared test was unable to demonstrate a significant statistical difference in a nominal distribution. Observationally, it also appears that if neonates were below the median, the distance to the median is greatest in the ES sample. Considering the difference to the median, a two-proportions z-test was performed, resulting in a statistically significant difference between the two samples with regard to all APGAR scores (*p* < 0.05, e.g., as shown in Table 5 for the 5-min APGAR).

## 4. Discussion

The study was able to demonstrate that a successful ECV permits a reduction in ES rates without risking a poorer neonatal outcome with respect to a successful vaginal birth. Under the aforementioned assumptions, an ECV trial alone was able to decrease the expected ES rate from 82.3% to 35.9% (RR 0.44), accepting an unplanned ES rate of 12.7%. These results appear to be in close concordance with large database analysis [12]. There is a 22% probability for an unplanned ES after a successful version and trial for a vaginal birth, which does not seem to be elevated compared to other large data samples [13]. This is, however, irrespective of parity, which has been shown to have a significant influence on the ECV outcome and subsequently on the mode of delivery [14]. To assess these findings more closely tailored to the population that delivers at the University Hospital in Duesseldorf, further data analysis is required. Specifically, we aim to assess the rate and outcome of unplanned caesarean sections after a successful ECV during trial of labour compared to a control that had a trial for a vaginal birth from a cephalic presentation without a previous ECV attempt. We surmise that, despite the small study sample, the findings appear to reflect results from other large studies and pooled result analyses. Concerning daily consultations for women with a non-cephalic presentation near term, a 78% probability of a vaginal birth can be expected, given a successful version, leaving a risk of approximately 22% for an unplanned caesarean section. These results apply to a population with a parity ratio of approximately 1:1 (nulliparous: multiparous). 

The study was able to provide scientific evidence regarding basic neonatal outcome parameters that can be helpful in consulting women near term with foetuses in a non-cephalic presentation. Based on the above data, women with a successful ECV can expect a neonatal birth outcome after a vaginal birth that is non-inferior to an alternative ES. In fact, the analysis indicates a slight advantage, in terms of APGAR score, for newborns after a vaginal birth. This may not be immediately obvious, as neonatal arterial pH and APGAR score appear to contradict each other. According to neonatal pH, newborns from a vaginal birth are significantly more acidic than those from an ES. However, neonatal APGAR scores from vaginally born foetuses demonstrate non-inferiority to the comparison sample. In fact, there is a trend that is supported by statistical significance that vaginally born neonates perform higher in all APGAR scores. Since an ES does not exert any physical stress on the foetus, it is inappropriate to compare neonatal pH values between an ES and vaginal births. Physical stress over a certain period results in a decrease in pH in most cases. The observation of higher umbilical cord blood pH in newborns after a caesarean section has been described previously [15,16]. Therefore, arterial umbilical cord blood pH is an inadmissible parameter for comparing neonatal wellbeing after an elective caesarean section to that after a vaginal birth. Nonetheless, some study groups have used arterial umbilical cord blood pH as a parameter to argue for the advantage of an ES [3]. In general, there is an ongoing debate on the prognostic value of routine blood gas analyses of umbilical cord blood in healthy newborns [17]. 

The most reliable information for assessing neonatal outcome is still the neonatal APGAR score with regards to this study. The analysis of APGAR score indicates an advantage for neonates born vaginally. This finding is consistent with the pooled results from the 2015 Cochrane analysis. However, it is important to emphasise that the data on this critical question are limited, primarily due to statistical heterogeneity and design limitations in the published studies [12]. 

Determining comparable population samples is crucial for addressing the aforementioned question. Parity has proven to be an influential factor [14]. Our study does not elucidate whether parity solely affects the outcome of an ECV attempt or if it also impacts birth outcomes. While our data suggest that nulliparous women with a successful ECV may have poorer neonatal outcomes, it is important to recognize the limitations inherent in our study design and sample size. The observed trend should be interpreted with caution due to the relatively small and specific sample population. Our sample size may not be sufficiently powered to generalise findings across all populations, particularly for nulliparous women. Therefore, this statement highlights an exploratory observation rather than a definitive conclusion. Secondly, modes of delivery must be strictly differentiated. In this context, arterial pH is only appropriate as a marker for neonatal birth outcomes within the same mode of delivery. Sorting study samples according to those basic parameters will supposedly yield more reliable data, particularly when study samples reach higher numbers in terms of participants. 

In summary, the findings of this retrospective study suggest that a vaginal birth after an ECV may have comparable neonatal outcomes to an elective caesarean section after an unsuccessful ECV. However, prospective studies are needed to confirm these results and draw definitive conclusions about noninferiority. On this basis, it is assumed that these results also apply to the comparison of a vaginal birth after a successful cephalic version to an ES without a previous version attempt. In this context, it must also be assumed that a trial for an ECV does not negatively impact the outcome of an ES from a non-cephalic presentation. In addition to neonatal benefits, a trial for an ECV leads to a considerable reduction in ES. Parity has a proven influence on the outcome of an ECV as well as on the mode of delivery. Nulliparous women, in particular, require more detailed counselling on the risks and benefits of an ECV attempt. In the authors’ opinion, these results should encourage women to opt for an ECV, especially if they are multiparous. As a result of the TBT trial and subsequent studies, an ES continues to be the dominant mode of delivery for term-pregnancies in a non-cephalic presentation in Germany. Implementing ECV in the daily clinical routine has proven to reduce the rate of ES.

## 5. Conclusions

Our study suggests that neonatal outcomes from a vaginal birth after a successful ECV are comparable to those from an elective caesarean section. Given the high success rate of ECV and favourable outcomes, especially among multiparous women, ECV should be considered a viable and safe option for managing non-cephalic near-term pregnancies. Healthcare providers are encouraged to routinely offer ECV to eligible pregnant women to potentially reduce the need for elective caesarean sections and their associated risks.

## Figures and Tables

**Table 1:** Absolute and relative proportions of the delivery mode.

	Mode of Delivery (MoD)				
ECV Attempt	Vaginal Birth (VB)	Elective Caesarean (ES)	Unplanned Caesarean	Σ	Proportion Given ECV Attempt
Yes	62	-	18	80	56.34%
No	11	51	-	62	43.66%
Σ	73	51	18	142	100%
Probability (MoD) After ECV Attempt	51.41%	35.92%	12.68%	100%	
Probability (MoD) * Without ECV Attempt	17.74%	82.26%	0.00%	100%	
Probalbility (VB|ECV yes)	77.50%	-			
Probability (ES|ECV no)	-	82.26%			

* Assumption: Numbers of MoD without an ECV are equal to the numbers of MoD with an unsuccessful ECV. ECV Attempt (Yes/No): Indicates whether an ECV attempt was made; Mode of Delivery (MoD): Shows the different types of delivery (vaginal birth, elective caesarean section (ES), unplanned caesarean section); Proportion Given ECV Attempt: The proportion of cases where an ECV attempt was made; Probability (MoD) with/without ECV Attempt: The probability of different modes of delivery given an ECV attempt; Probability (VB|ECV yes): The probability of a vaginal birth given a successful ECV; Probability (ES|ECV no): The probability of an elective caesarean section given an unsuccessful ECV.

**Table 2:** Basic maternal characteristics within its mode of delivery. N = Sample size; SD = Standard Deviation; SEM = Standard Error of the Mean; *p*-value calculated with an independent samples *t*-test. There is a statistically significant two-year difference in maternal age, most likely because of the difference in parity. The remaining maternal characteristics appear to be homogenous.

Basic Maternal Characteristics	Mode of Delivery	N	Mean	SD	SEM	*p*-Value
Age in years	Vaginal birth after ECV	62	33.35	5.18	0.66	0.029
	ES after unsuccessful ECV	51	31.31	4.47	0.63	
Weight in kilograms	Vaginal birth after ECV	61	65.16	12.58	1.61	0.301
	ES after unsuccessful ECV	51	67.50	10.92	1.53	
Height in metres	Vaginal birth after ECV	62	1.67	0.07	0.01	0.463
	ES after unsuccessful ECV	51	1.68	0.07	0.01	
Body mass index	Vaginal birth after ECV	61	23.49	4.09	0.52	0.413
	ES after unsuccessful ECV	51	24.14	4.21	0.59	

**Table 3:** Describing the proportions of a vaginal birth and ES after an unsuccessful ECV dependent on parity. Nulliparous women are significantly less likely (Pearson Chi-squared test) to succeed in a vaginal birth than multiparous (*p* ≥ 1) women.

Mode of Delivery	*p*-Value		Parity		Total
			Nulliparous	Multiparous	
Vaginal Birth after ECV	<0.001	Count	21	41	62
ES after unsuccessful ECV		Count	38	13	51
Total		Count	59	54	113

**Table 4:** Basic neonatal characteristics: Mean gestational age in the vaginal-birth group differs significantly from the ES group, resulting also in a significantly higher birthweight in the vaginal-birth group. Also, the standard deviation of 503.7 g is greater in the vaginal-birth group than in the ES group (368.5 g). Neonatal length and head circumference are similar in both study groups. Statistical differences were tested using an independent Samples *t*-Test.

Basic Neonatal Characteristics	Mode of Delivery	N	Mean	SD	SEM	*p*-Value
Gestational Age at Birth (Days)	Vaginal Birth after ECV	61	276.13	9.96	1.28	<0.001
	ES after unsuccessful ECV	51	269.45	5.42	0.76	
Weight at Birth (Grams)	Vaginal Birth after ECV	60	3457.08	503.69	65.03	0.025
	ES after unsuccessful ECV	50	3267.40	368.51	52.11	
Length at Birth (Centimetres)	Vaginal Birth after ECV	60	51.11	2.55	0.33	0.110
	ES after unsuccessful ECV	50	50.37	2.19	0.31	
Head Circumference at Birth (Centimeters)	Vaginal Birth after ECV	60	35.11	1.26	0.16	0.706
	ES after unsuccessful ECV	50	35.20	1.39	0.20	

**Table 5:** Test statistic for a lower-tailed “two-proportions z-test” for the 5-minute APGAR score. The null-hypothesis assumes a greater or equal difference to the median for neonates born vaginally compared to ES after an unsuccessful ECV. A *p*-value < 0.05 allows the rejection of null and the acceptation that neonates after an ES deviate from the median 10 significantly more than neonates after a vaginal birth. 1- and 10-minute APGAR scores also show statistical significance in the above test statistic.

Lower Tailed Two-Proportion z-Test for 5-Minute APGAR Score							
Mode of Delivery	n	Risk Units	p̂	Pooled p̂	z-Test	Critical z-Value	*p*-Value
Vaginal Birth after ECV	61	4	0.007	0.017	−2.950	−1.645	0.002
ES after unsuccessful ECV	51	15	0.029				

**Table 6:** Comparing postnatal arterial pH after a vaginal birth to an ES after an unsuccessful ECV. *p*-value for equality of variances was tested using Levene’s test. *p*-Value for equality of means was tested using Student’s-*t*-Test. Results demonstrate a significant statistical difference in mean and variance between both samples.

Postnatal Arterial pH						
Mode of Delivery	N	Mean	SD	SEM	*p*-Value for Equality of Variances	*p*-Value for Equality of Means
Vaginal Birth after ECV	58	7.262	0.089	0.012	<0.001	<0.001
ES after unsuccessful ECV	50	7.316	0.051	0.007		

## Data Availability

The datasets generated and/or analysed during the current study are not publicly available due to reasons of data protection but are available from the corresponding author on reasonable request.
